# Adverse childhood experiences and other risk factors associated with adolescent and young adult vaping over time: a longitudinal study

**DOI:** 10.1186/s12889-021-12477-y

**Published:** 2022-01-14

**Authors:** Janique Fortier, Tamara Taillieu, Samantha Salmon, Ashley Stewart-Tufescu, Isabel Garcés Davila, Harriet L. MacMillan, Jitender Sareen, Lil Tonmyr, Marni Brownell, Nathan C. Nickel, Tracie O. Afifi

**Affiliations:** 1 Department of Community Health Sciences, University of Manitoba, Winnipeg, Manitoba Canada; 2 Faculty of Social Work, University of Manitoba, Winnipeg, Manitoba Canada; 3 Departments of Psychiatry and Behavioural Neurosciences, and of Pediatrics, McMaster University, Hamilton, Ontario Canada; 4 Department of Psychiatry, University of Manitoba, Winnipeg, Manitoba Canada; 5 Public Health Agency of Canada, Ottawa, Ontario Canada; 6 Manitoba Centre for Health Policy, University of Manitoba, Winnipeg, Manitoba Canada; 7 Departments of Community Health Sciences and Psychiatry, University of Manitoba, S113-750 Bannatyne Avenue, Winnipeg, Manitoba R3E 0W5 Canada

**Keywords:** Vaping, E-cigarette, Electronic vaping product, Adverse childhood experiences (ACEs), Parental smoking and vaping, Mental disorders

## Abstract

**Background:**

Vaping among adolescents and young adults is a significant public health concern worldwide. Understanding which risk factors are associated with vaping is important to help inform evidence-based prevention and intervention strategies. There are several gaps in the current literature examining these associations such as limited longitudinal research. We examined the association between parental smoking/vaping, adolescent sex, mental disorders in adolescence, 13 adverse childhood experiences (ACEs) and a) any vaping and b) course of vaping across two time points among adolescents and young adults.

**Methods:**

Data were from Waves 1 and 2 of the longitudinal Well-Being and Experiences Study (The WE Study) in Manitoba, Canada which collected data from a community sample of adolescents (14 to 17 years) and their parent/caregiver in Wave 1 in 2017–18 and the adolescents/young adults only in Wave 2 in 2019. A total of 752 adolescents/young adults (72.4% of the original cohort) completed both waves of the study. Binary and multinomial logistic regressions were conducted to understand the relationship between the 16 risk factors and the two vaping outcomes.

**Results:**

Vaping prevalence was 45.5% for any vaping, 2.7% for Wave 1 vaping only, 19.7% for new onset Wave 2 vaping, and 21.2% for vaping at both waves. After adjusting for covariates, the majority of risk factors examined were associated with any adolescent or young adult vaping, including: parental smoking or vaping, emotional abuse, emotional neglect, exposure to verbal intimate partner violence, household substance use, household mental illness, parental separation/divorce, parental problems with police, foster care or contact with a child protective organization, an unsafe neighbourhood, and peer victimization. The majority of these risk factors, as well as adolescent mental health and parental gambling, were associated with different courses of vaping across the two time points.

**Conclusions:**

The findings emphasize the need for early vaping prevention and identified several ACEs and other factors that were associated with adolescent and young adult vaping and course of vaping. These identified ACEs and risk factors can help inform programs, strategies, and potential groups to target for vaping interventions.

## Background

The rapidly growing use of nicotine electronic vaping products, commonly referred to as electronic cigarettes, e-cigarettes, and vaping, among youth is a significant concern among health agencies globally, with some public health officials referring to it as an epidemic [1–4]. As an example of the harm associated with e-cigarettes, the significant increase in their use was identified as the main driver in increased tobacco use among youth in 2017–2018, based on a United States (US) nationally representative sample of grades 6 to 12 students [5]. This essentially erased the progress made in tobacco reduction from 2011 to 2017; e-cigarettes have become the most commonly used tobacco product among middle and high school students since 2014 [5, 6]. In Canada and the US in 2018, one study noted the highest increase in vaping prevalence documented thus far (increase of five and six percentage points, respectively) among adolescents (ages 16 to 19 years) [7]. Increases in e-cigarette use have also been noted among young adults (ages 18 to 24 years) in the U.S. whereas the prevalence in older age groups has remained the same or even declined from 2014 to 2018 [8]. There have been frequent calls to action to protect youth by preventing vaping initiation and helping them discontinue use [2].

Understanding which factors are associated with youth vaping is important to inform evidence-based approaches to prevention and cessation of e-cigarette use among youth. In recent years, a growing literature shows that several risk factors, including sociodemographic variables, substance use (including tobacco use), psychological factors, household tobacco use, relationship with parents, are associated with various vaping indicators in adolescence including susceptibility to initiation (i.e., vaping intention and willingness), initiation, lifetime use, current use, and dual use with traditional tobacco products [9–14]. For example, in a longitudinal study among a nationally representative sample of U.S. adolescents ages 12 to 17 years, the authors found past-year high externalizing problems, alcohol use, and older age to be associated with subsequent initiation of e-cigarette and dual (e-cigarette and combustible cigarette) use [14]. The same study also found past-year internalizing problems associated with subsequent initiation of e-cigarette use, past year marijuana use to be significantly associated with dual (e-cigarette and combustible cigarette) use, and African American students to be less likely to initiate e-cigarette and dual use compared to White adolescents [14]. A review of recent literature on sex and gender differences in e-cigarette use among American adolescents found limited and mixed findings; some studies reported e-cigarette use to be more prevalent among males, and other studies examined reported no sex and gender differences [15].

The relationship between various adverse childhood experiences (ACEs, [16]) and more traditional tobacco or nicotine use has been widely studied in the literature finding consistent associations between ACEs and tobacco use [17–21], but less is known about the association between ACEs and e-cigarette use. A few studies have examined the relationship between ACEs and vaping in young adult and adult samples. In a U.S. urban community sample of young adults aged 18 to 21 years, the authors found physical abuse, sexual abuse, emotional abuse, emotional neglect, and physical neglect to be associated with greater likelihood of lifetime e-cigarette use [22]. In a sample of adult Australian women (ages 19–26 years), the authors found psychological abuse, physical abuse, sexual abuse, household substance abuse, witnessing domestic violence, household mental illness, and parental separation or divorce (but not an incarcerated household member) were significantly associated with past-year and lifetime e-cigarette use [23]. Another study involving adults in central Florida, found an association between a history of four or more ACEs and e-cigarette use [24].

A few studies have examined the relationship between ACEs and vaping in adolescent samples [25–27]. One study conducted with a sample of students ages 13 to 18 years in Bangkok, Thailand, found several ACEs to be associated with higher odds of lifetime e-cigarette use [25]. A study using data from Wave 1 of the Well-Being and Experiences (WE) Study (same data used in the current study) conducted in Manitoba, Canada, examined the relationship between an expanded list of ACEs [26] and past 30-day electronic vapor product use [27]. The findings showed a significant association with emotional abuse, exposure to verbal intimate partner violence (IPV), parental separation or divorce, household mental illness, household substance use, parental problem gambling, and parental problems with police (but not emotional neglect, spanking, foster care placement or contact with a child protective organization (CPO), an unsafe neighbourhood, poverty in the unadjusted model, and peer victimization without other ACEs) [27]. Furthermore, a few studies (both young adult and adolescent samples) have also found graded relationships between the number of ACEs and vaping outcomes [23, 25, 28]. Among studies that examined emotional abuse, physical abuse, sexual abuse, and parental separation or divorce, they have all shown an association with vaping outcomes [22, 23, 27]. For several other ACEs, there have been mixed findings [22, 23, 25, 27].

While there is an emerging and growing literature examining risk factors associated with vaping, important gaps remain. Small community samples, along with use of different measures, various age groups, and varied vaping outcomes may, in part, explain the mixed findings noted in the literature, particularly as they relate to sex and gender [15] and ACEs. A notable gap in the literature is the lack of longitudinal studies. One of the few longitudinal studies published examined the relationship between internalizing and externalizing problems and initiation of e-cigarette use, but not how vaping use changed over time [14]. Little is known about what factors are associated with trajectories of vaping use over time. One longitudinal study that examined the change in frequency of e-cigarette use from spring of grade 8 (about 14 years old) to spring of grade 9 (about 15 years old) only examined the relationship to sex and gender, ethnicity, and use of other substances and found greater lifetime substance use in grade 8 and current substance use in grade 9 among accelerating users [29].

Given the limitations noted above, the objectives of the current study were to examine what risk factors, including self-reported ACEs, were associated with a) any adolescent and young adult vaping and b) adolescents’ and young adults’ course of vaping across two time points using longitudinal data.

## Methods

### Data and sample

Data were from Wave 1 (W1; July 2017–October 2018) and Wave 2 (W2; October 2019–January 2020) of the longitudinal Well-Being and Experiences (WE) Study in Manitoba, Canada. W1 collected data from matched parent/caregiver (*N* = 1000, 96.1% biological parents) and adolescent (*N* = 1000; 14 to 17 years) dyads who self-completed computerized questionnaires privately at a research facility in separate rooms. The W1 adolescent inclusion criteria included that the adolescent: 1) be between the ages of 14 and 17 years; 2) have a parent or guardian (not foster parent) that was willing to participate in the study; 3) have the ability to communicate well in English verbally and in writing; and 4) reside in Manitoba. Participants were recruited via random digit dialing (21%) and convenience sampling (i.e., referrals and community advertisements; 79%). Using random digit dialing, 83% of those contacted were interested in participating; however, only 3% had an adolescent aged 14 to 17 years old making them eligible to participate. Among those eligible, 63% provided consent and participated in the study. More details on W1 data collection and sample have been published elsewhere [26]. Data collection for W2 was completed anywhere via an online questionnaire on personal devices by W1 adolescents who provided consent to be contacted for future studies. The sample of adolescents and young adults who participated in both W1 and W2 of the WE Study (*n* = 752, 72.4% of the original adolescent cohort, now 15 to 20 years) did not differ significantly from the sample who only participated in W1 on age, sex, emotional abuse, exposure to verbal IPV, household mental illness, parental problem with police, spanking/slapping, parental gambling, unsafe neighbourhood, peer victimization, and self-rated mental health. Respondents missing from W2 were more likely to have multiple ethnicities (belonging to more than one of the following racial, ethnic, or cultural groups: White, South Asian, Southeast Asian, West Asian, Chinese, Korean, Japanese, Black, Filipino, Latin American, Arab, First Nations, Metis, Inuit, other) or an ethnic identity other than ‘White’, experienced emotional neglect, household substance abuse, divorced parents, been in foster care or in contact with a CPO, and lived in poverty. They were also less likely to have a baseline household income of $50,000 and higher.

### Measurement

#### Independent variables

All independent variables were assessed in W1 and included: parental smoking or vaping, adolescent sex (female vs. male), adolescent mental disorders, and 13 ACEs. Parents/caregivers were categorized as having smoked or vaped if they indicated smoking a cigarette or using an electronic vapor product one or more days (vs. 0 days or never tried) in the past 30 days. Adolescent mental disorders were assessed by asking if they currently had a long-term health condition that had lasted or expected to last 6 months or more and had been diagnosed by a medical doctor or other health care professional. Adolescents were categorized as having a mental disorder if they responded *yes* to any of the following: depression, bipolar disorder, anxiety disorder, obsessive-compulsive disorder, posttraumatic stress disorder, attention-deficit/hyperactivity disorder or attention deficit disorder, eating disorder, alcohol problems, drug problems, autism or Asperger’s, learning or developmental disability (other than cerebral palsy), oppositional defiant disorder, or conduct disorder.

The current study used an expanded list of 13 ACEs [26], including: emotional abuse, emotional neglect, exposure to verbal IPV, spanking, household substance use, household mental illness, parental separation/divorce, parental problems with police, parental gambling, foster care placement or contact with CPO, poverty, unsafe neighbourhood, and peer victimization. The Childhood Trauma Questionnaire was used to assess emotional neglect using five items adapted to the present tense [30]. Items from or adapted from the Childhood Experiences and Violence Questionnaire were used to assess exposure to verbal IPV and spanking [31]. Household substance use, household mental illness, parental separation/divorce, and parental problems with police were assessed using items adapted from the ACEs Study [16]. Adolescents were categorized as having experienced:

1. emotional abuse - if they reported having a parent or other adult living in their home say hurtful or mean things to them *once a month* or more (vs. *several times a year* or less) in the past 12 months;
2. exposure to verbal IPV - if they reported seeing or hearing adults say hurtful or mean things to another adult in their home in the past 12 months *once a month* or more (vs. *several times a year* or less);
3. spanking - if they reported remembering an adult spanking them with their hand on their bottom before the age of 10 years *two to three times a year* or more (vs. *once a year* or less);
4. household substance use - if they responded *yes* to a parent or other adult living in their home ever having: a) problems with alcohol or spending a lot of time drinking or being hungover and/or b) problems with drugs;
5. parental separation or divorce of their biological parents - if they responded *yes*;
6. household mental illness – if responded *yes* to a parent or other adult living in their home ever had mental health issues like depression or anxiety;
7. parental (or other adult living in their home) problems with police - if they responded *yes*;
8. parental (or other adult living in their home) gambling – if they responded *yes*;
9. foster care or CPO contact - if they responded *yes* to having ever: a) seen or talked to anyone from a CPO due to difficulties at home and/or b) ever been placed in a foster home or group home by the Child and Family Services;
10. poverty - if they responded *sometimes* or more (vs. *rarely* or *never*) on one or both of the following items: a) How often does your family run out of money or find it hard to pay for rent or mortgage on your house? and b) How often does your family run out of money or find it hard to pay for basic necessities like food or clothing?
11. peer victimization - if they indicated experiencing any of the following seven forms of peer victimization by a friend, peer, kid at school, or other young person (not an adult or sibling) *once a month* or more (vs. *7 to 11 times a year* or less) in the past 12 months: a) pushed you, shoved you, tripped you, or spit on you; b) bullied, picked on you, or said mean things about you, or threatened you through texting or the Internet (e.g., posted something on Facebook or other social media, or sent texts or emails), and/or the following in person or behind your back (excluding texting, email, social medial, or online posting or communications); c) made fun of you, called you names, or insulted you; d) spread rumours about you; e) said something bad about your race, culture, or religion; f) said something bad about your sexual orientation or gender identity; and g) said something bad about your body, shape, size, or appearance in person or behind your back;
12. an unsafe neighbourhood - if they responded *strongly disagree* or *disagree* (vs. *neither agree nor disagree* or stronger agreement) to the statement: I feel safe in my community.

Adolescents were not asked about physical abuse, sexual abuse, and physical neglect in W1 due to mandatory reporting laws for this age group (younger than 18 at the time). While these were asked of participants 18 years and older at W2, for consistency and to maintain the longitudinal nature of the data, we did not include them in the present study.

#### Adolescent and young adult vaping

In W1, adolescents were asked during the past 30 days, on about how many days did you use an electronic vapor product (such as e-cigarettes, e-cigars, vape pipes, vaping pens)? In W2, adolescents and young adults were asked three vaping questions: whether they had ever used an electronic vapor product for nicotine, whether they had used in the past 12 months, and whether they had used in the past 30 days. The W2 survey used skip logic such that respondents who indicated no lifetime vaping at W2 would not be asked subsequent vaping questions and those who indicated no past 12 months use at W2 were not asked the past 30 days vaping question. Two vaping variables were created: first, an any vaping variable (*yes/no*) was created where adolescents and young adults indicated using an electronic vapor product once or more on any of the four W1 or W2 questions. Second, a four-level vaping across time variable was created: no vaping at both time points, W1 only, new onset W2 vaping, and vaping at both W1 and W2. Respondents were considered to have vaped at W2 if they had indicated having used an electronic vaping product in the past 12 months or in the past 30 days. The W2 lifetime vaping question was only used to include respondents who had indicated ‘no’ lifetime use and therefore were not asked subsequent vaping questions due to the skip logic of the survey. Respondents who indicated vaping in the past 30 days at W1, but no to lifetime vaping at W2 were coded as missing in the across time variable (*n* = 25).

#### Covariates

Statistical analyses adjusted for three sociodemographic covariates, including parent reported household income ($49,000 or less, $50,000 to $99,999, $100,000 to $149,999, and $150,000 or more), parental marital status (married or common-law; separated, divorced, or widowed; and never married), and adolescent age at W1 (14 to 17 years).

### Statistical analysis

First, descriptive statistics were computed to determine the prevalence of adolescent and young adult vaping (i.e., any vaping, vaping across time) and any vaping by parental and adolescent risk factors and sociodemographic variables. Second, logistic regressions were computed to examine the relationship between parental and adolescent risk factors and any (W1 or W2) adolescent and young adult vaping unadjusted and adjusted for sociodemographic covariates. Third, unadjusted and adjusted multinomial logistic regressions were computed to examine the relationship between parental and adolescent risk factors and adolescent and young adult vaping across time (W1 to W2) using the four-level categorical variable described above.

## Results

The sample prevalence of any adolescent and young adult vaping (W1 or W2) was 45.5% (*n* = 331). Using the vaping across time variable, a total of 2.7% of adolescents reported vaping at W1 only (*n* = 19), 19.7% at W2 only (*n* = 138), and 21.2% at both W1 and W2 (*n* = 149). The prevalence of any adolescent and young adult vaping by parental and adolescent risk factors and sociodemographic variables are presented in Table 1 and by ACEs in Table 2.

**Table 1:** Prevalence of any Wave 1 or Wave 2 vaping by parental and adolescent risk factors and sociodemographic variables

	Never Vaping % (n)	Any Vaping % (n)
**Household income**		
$49,999 or less	53.7 (66)	46.3 (57)
$50,000 to $99,999	53.3 (136)	46.7 (119)
$100,000 to $149,999	57.7 (98)	42.4 (72)
$150,000 or more	52.7 (77)	47.3 (69)
No response	54.8 (17)	45.16 (14)
**Parental marital status**		
Married or common-law	56.4 (333)	43.7 (258)
Separated, divorced, or widowed	50.5 (54)	49.5 (53)
Never married	25.9 (7)	74.1 (20)
**Parental smoking or vaping**		
Yes	40.4 (40)	59.6 (59)
No	56.4 (349)	43.6 (270)
**Adolescent sex**		
Male	55.2 (186)	44.8 (151)
Female	53.8 (208)	46.3 (179)
**Adolescent age**		
14	62.2 (140)	37.8 (85)
15	54.9 (107)	45.1 (88)
16	44.9 (83)	55.1 (102)
17	54.1 (66)	45.9 (56)
**Adolescent mental disorder**		
Yes	48.7 (76)	51.3 (80)
No	56.1 (289)	43.9 (226)

**Table 2:** Prevalence of any Wave 1 or Wave 2 vaping by adolescent adverse childhood experiences

	Never Vaping % (n)	Any Vaping % (n)
Emotional abuse		
Yes	41.7 (60)	58.3 (84)
No	57.6 (307)	42.4 (226)
Emotional neglect		
Yes	36.2 (17)	63.8 (30)
No	56.0 (374)	44.0 (294)
Exposure to verbal IPV		
Yes	41.1 (62)	58.9 (89)
No	57.5 (293)	42.6 (217)
Spanking		
Yes	54.3 (115)	45.8 (97)
No	54.7 (260)	45.3 (215)
Household substance use		
Yes	40.4 (40)	59.6 (59)
No	58.1 (330)	41.9 (238)
Household mental illness		
Yes	48.2 (106)	51.8 (114)
No	61.0 (230)	39.0 (147)
Parental separation/ divorce		
Yes	46.6 (82)	53.4 (94)
No	57.6 (293)	42.4 (216)
Parental problems with police		
Yes	39.3 (24)	60.7 (37)
No	56.2 (337)	43.8 (263)
Parental gambling		
Yes	33.3 (7)	66.7 (14)
No	54.7 (366)	45.3 (303)
Foster care or contact with CPO		
Yes	39.5 (32)	60.5 (49)
No	56.6 (356)	43.4 (273)
Poverty		
Yes	53.6 (59)	46.4 (51)
No	53.3 (276)	46.7 (242)
Unsafe neighbourhood		
Yes	33.3 (10)	66.7 (20)
No	55.2 (382)	44.8 (310)
Peer victimization		
Yes	44.1 (71)	55.9 (90)
No	56.5 (286)	43.5 (220)

*IPV* Intimate partner violence, *CPO* Child protective organization

Table 3 presents the associations between parental and adolescent risk factors and any adolescent and young adult vaping (W1 or W2). The following risk factors were significantly associated with increased odds of any vaping: parental smoking or vaping, emotional abuse, emotional neglect, exposure to verbal IPV, household substance use, household mental illness, parental separation/divorce (unadjusted model only), parental problems with police, foster care or contact with CPO, unsafe neighbourhood, and peer victimization.

**Table 3:** Parental and adolescent risk factors associated with any Wave 1 or Wave 2 adolescent and young adult vaping

	OR (95% CI)	AOR (95% CI)
Parental smoking or vaping	1.91 (1.24, 2.94)**	1.81 (1.14, 2.85)*
Adolescent sex (Female vs. Male)	1.06 (0.79, 1.42)	1.01 (0.74, 1.36)
Adolescent mental disorders	1.35 (0.94, 1.93)	1.25 (0.86, 1.82)
Emotional abuse	1.90 (1.31, 2.76)***	1.92 (1.31, 2.82)***
Emotional neglect	2.24 (1.21, 4.15)**	2.15 (1.14, 4.04)*
Exposure to Verbal IPV	1.94 (1.43, 2.80)***	1.92 (1.31, 2.80)***
Spanking	1.02 (0.74, 1.41)	1.03 (0.74, 1.45)
Household substance use	2.05 (1.32, 3.16)***	1.92 (1.22, 3.01)**
Household mental illness	1.68 (1.20, 2.35)**	1.56 (1.09, 2.23)*
Parental separation/divorce	1.55 (1.10, 2.19)*	1.50 (0.92, 2.44)
Parental problems with police	1.98 (1.15, 3.38)*	1.91 (1.09, 3.35)*
Parental gambling	2.42 (0.96, 6.06)	1.89 (0.73, 4.89)
Foster care or contact with CPO	2.00 (1.24, 3.20)**	1.90 (1.15, 3.14)*
Poverty	0.99 (0.65, 1.49)	0.96 (0.61, 1.51)
Unsafe neighbourhood	2.46 (1.14, 5.34)*	2.25 (1.01, 5.01)*
Peer victimization	1.65 (1.15, 2.36)**	1.70 (1.17, 2.46)**

Regression models compared to never vaping

*OR* Odds ratio, *CI* Confidence interval, *AOR* Adjusted odds ratio, *IPV* Intimate partner violence, *AOR* Adjusting for parental marital status, household income, and adolescent age

*p ≤ .05, ** *p* ≤ .01, *** *p* ≤ .001

Table 4 presents the associations between parental and adolescent factors and adolescent and young adult vaping across time (W1 to W2). Parental smoking or vaping (in both the unadjusted and adjusted models) was associated with significantly increased odds of W1 only and new onset (W2) vaping. Household substance use was associated with increased odds of W1 only vaping (unadjusted model only) and both W1 and W2 vaping (both unadjusted and adjusted models). Emotional neglect, foster care or contact with CPO, and unsafe neighbourhood were associated with significantly increased odds of new onset (W2) vaping in both the unadjusted and adjusted models. Emotional abuse, exposure to verbal IPV, and household mental illness were associated with significantly increased odds of new onset (W2) vaping and both W1 and W2 vaping (in both unadjusted and adjusted models). Adolescent mental disorders (unadjusted model only), parental separation or divorce (unadjusted model only), parental gambling (both unadjusted and adjusted models), and peer victimization (both unadjusted and adjusted models) were associated with significantly increased odds of both W1 and W2 vaping.

**Table 4:** Parental and adolescent risk factors associated with course of vaping across time (Wave 1 to Wave 2)

	W1 Only Vaping	New Onset W2 Vaping	Both W1 and W2 Vaping
Parental smoking or vaping			
OR (95% CI)	6.35 (2.41, 16.70)***	1.93 (1.12, 3.32)*	1.29 (0.72, 2.30)
AOR (95% CI)	5.31 (1.81, 15.53)**	1.86 (1.05, 3.30)*	1.27 (0.68, 2.37)
Adolescent sex (Female vs. Male)			
OR (95% CI)	0.65 (0.26, 1.65)	1.11 (0.75, 1.65)	1.04 (0.71, 1.51)
AOR (95% CI)	0.56 (0.21, 1.48)	1.10 (0.74, 1.65)	0.91 (0.62, 1.36)
Adolescent mental disorders			
OR (95% CI)	2.22 (0.84, 5.83)	1.06 (0.65, 1.74)	1.63 (1.05, 2.53)*
AOR (95% CI)	1.69 (0.60, 4.73)	1.11 (0.67, 1.84)	1.40 (0.88, 2.22)
Emotional abuse			
OR (95% CI)	1.10 (0.31, 3.93)	1.87 (1.16, 3.00)**	2.09 (1.32, 3.31)**
AOR (95% CI)	1.11 (0.30, 4.12)	1.92 (1.19, 3.12)**	2.06 (1.28, 3.31)**
Emotional neglect			
OR (95% CI)	2.93 (0.62, 13.87)	2.70 (1.31, 5.58)**	1.45 (0.63, 3.32)
AOR (95% CI)	2.19 (0.42, 11.41)	2.64 (1.25, 5.54)*	1.29 (0.55, 3.07)
Exposure to verbal IPV			
OR (95% CI)	1.35 (0.43, 4.24)	1.82 (1.13, 2.93)*	2.07 (1.31, 3.26)**
AOR (95% CI)	1.30 (0.40, 4.19)	1.87 (1.15, 3.04)*	2.01 (1.25, 3.22)**
Spanking			
OR (95% CI)	0.45 (0.13, 1.59)	0.98 (0.63, 1.51)	1.09 (0.72, 1.66)
AOR (95% CI)	0.43 (0.12, 1.55)	0.99 (0.63, 1.54)	1.11 (0.72, 1.72)
Household substance use			
OR (95% CI)	3.17 (1.08, 9.37)*	1.48 (0.82, 2.66)	2.48 (1.48, 4.17)***
AOR (95% CI)	2.33 (0.73, 7.42)	1.45 (0.79, 2.64)	2.44 (1.42, 4.19)***
Household mental illness			
OR (95% CI)	2.48 (0.88, 7.02)	1.65 (1.04, 2.59)*	1.85 (1.21, 2.81)**
AOR (95% CI)	1.57 (0.50, 4.90)	1.73 (1.07, 2.81)*	1.63 (1.03, 2.56)*
Parental separation/divorce			
OR (95% CI)	1.62 (0.55, 4.81)	1.48 (0.94, 2.32)	1.71 (1.12, 2.63)*
AOR (95% CI)	0.25 (0.03, 1.92)	1.63 (0.88, 3.01)	1.77 (0.97, 3.25)
Parental problems with police			
OR (95% CI)	2.01 (0.43, 9.34)	1.91 (0.97, 3.78)	1.76 (0.89, 3.46)
AOR (95% CI)	1.45 (0.30, 7.08)	1.87 (0.92, 3.78)	1.78 (0.88, 3.62)
Parental gambling			
OR (95% CI)	–	1.23 (0.31, 4.81)	3.87 (1.45, 10.38)**
AOR (95% CI)	–	0.84 (0.20, 3.62)	3.18 (1.13, 8.90)*
Foster care or contact with CPO			
OR (95% CI)	1.31 (0.29, 5.92)	2.50 (1.41, 4.42)**	1.74 (0.96, 3.15)
AOR (95% CI)	0.65 (0.13, 3.28)	2.67 (1.45, 4.93)***	1.62 (0.86, 3.06)
Poverty			
OR (95% CI)	2.13 (0.71, 6.35)	1.02 (0.59, 1.77)	0.83 (0.48, 1.42)
AOR (95% CI)	1.36 (0.41, 4.44)	0.96 (0.53, 1.74)	0.93 (0.51, 1.68)
Unsafe neighbourhood			
OR (95% CI)	4.78 (0.97, 23.61)	2.67 (1.06, 6.70)*	1.88 (0.70, 5.04)
AOR (95% CI)	2.99 (0.52, 17.21)	2.62 (1.02, 6.75)*	1.87 (0.67, 5.22)
Peer victimization			
OR (95% CI)	2.35 (0.89, 6.18)	1.53 (0.96, 2.45)	1.71 (1.10, 2.67)*
AOR (95% CI)	2.52 (0.92, 6.88)	1.52 (0.94, 2.47)	1.84 (1.15, 2.93)*

Multinomial regression models compared to never vaping

*W1* Wave 1, *W2* Wave 2, *OR* Odds ratio, *CI* Confidence interval, *AOR* Adjusted odds ratio, *AOR* Adjusting for parental marital status, household income, and adolescent age

*p ≤ .05, ** *p* ≤ .01, *** *p* ≤ .001

## Discussion

The current study identified several unique findings related to adolescent vaping and the risk factors associated with different trajectories. First, the sample prevalence of any vaping in the current study was 45.5% – higher than Canadian estimates of adolescent and young adult vaping in 2017 (23 and 29%, respectively) [32]. Differing populations, years of data collection, and study design (i.e., cross sectional vs. longitudinal) may, in part, explain the discrepancy. The sample in the current study is not representative so the findings may not be generalizable to other populations. Second, the vast majority (88.6%) of adolescents who had vaped at W1 (*n* = 168) continued to do so as older adolescents or young adults at W2. Third, the majority of ACEs and risk factors studied were associated with any adolescent or young adult vaping, including: parental smoking or vaping, emotional abuse, emotional neglect, exposure to verbal IPV, household substance use, household mental illness, parental separation/divorce (unadjusted model only), parental problems with police, foster care or contact with a CPO, an unsafe neighbourhood, and peer victimization. Fourth, the majority of these ACEs and risk factors (associated with any adolescent or young adult vaping), and the addition of adolescent mental health and parental gambling, were associated with different courses of vaping across the two time points (some in the unadjusted models only).

The findings of the current study are generally consistent with one or more published studies in the literature [10, 14, 15, 22, 23, 25, 27, 33], with the exception of foster care or contact with a CPO, an unsafe neighbourhood, and peer victimization, which have not been found to be associated with vaping outcomes in a previous study using W1 of the WE Study data [27]. Importantly, the current study extends knowledge by examining the associations with adolescent and young adult courses of vaping over two time points using longitudinal data. Some of the mixed findings in the literature, particularly as they relate to ACEs, along with our findings that ACEs and other risk factors are associated with different courses of vaping over time, emphasize the importance of taking a more nuanced approach with greater specificity when examining risk factors associated with vaping among adolescents and young adults. For example, in our findings, adolescent mental health problems and parental gambling were not significantly associated with the ‘any vaping’ variable; however, these risk factors were significantly associated with vaping at both W1 and W2 (mental health problems in the unadjusted model only).

The finding that the vast majority of adolescents who reported vaping at W1 also reported vaping at W2 highlights the importance of early prevention of vaping initiation. The factors associated with vaping at both waves identified in the current study may help inform prevention strategies and identify target population groups. These ACEs and other risk factors included adolescent mental disorders (unadjusted model only), emotional abuse, exposure to verbal IPV, household substance use, household mental illness, parental separation/divorce (unadjusted model only), parental gambling, and peer victimization. Although parental problems with police, foster care or contact with CPO, and an unsafe neighbourhood were not significantly associated with vaping at both waves, each association had moderate effect sizes, similar to other factors that were studied. It is possible that the lack of statistical significance is a type II error due to rare occurrences of these events and power issues due to small sample size. Future studies with larger sample sizes should examine these relationships further. The findings of the current study suggest that any of these significant risk factors could be targeted to inform prevention strategies and identify target populations at various points in adolescents and young adults’ vaping trajectories. However, these findings do not provide a clear understanding of which adolescents quit vaping after W1. More research is needed to understand protective factors that may be associated with vaping cessation as well as prevention.

This study has several limitations. First, causation cannot be inferred; the associations with the “any vaping” variable are cross sectional and, among those who reported vaping in the past 30 days in W1, it is unknown when initiation first occurred and whether it may have preceded the factors studied. Second, the adolescent and young adult vaping questions changed slightly from W1 to W2. In W1, respondents were asked about past 30-day use only and the question did not specify for nicotine use while in W2 they were asked specifically about vaping for nicotine use for lifetime, 12 months, and past 30 days. The difference in wording may result in an overestimate of the number of adolescents vaping nicotine in W1 as it may include those who used electronic vaping products for other substances (e.g., nicotine free or marijuana). However, this is likely a very small proportion of respondents. In one Canadian survey of 3034 regular e-cigarette users aged 16 to 24 years, the vast majority of respondents (91.3%) indicated using electronic vaping products containing nicotine and only 1.2 to 3.8% were currently vaping cannabis products [34]. Furthermore, the difference in timeframes in the current study between waves may have also influenced the results. For example, the number of respondents who used an electronic vaping product in W1 may be underestimated due to the shorter timeframe (past 30 days) compared to W2 (past 12 months and/or past 30 days). When parents were asked about electronic vaping product use in W1, the question also did not specify for nicotine use. Third, some of the measures may be subject to recall bias. Fourth, due to mandatory child abuse reporting laws, we were unable to ask adolescents about their experiences of the following ACEs: physical abuse, sexual abuse, and physical neglect. Fifth, the use of single item questions to measure many of the ACEs in the current study, while common in the literature, has been critiqued as simplistic [35]. Sixth, attrition from W1 to W2 due to the longitudinal nature of the study may have biased the results. Finally, the community sample recruited primarily by non-random recruitment methods may not be representative and therefore the findings may not be generalizable to other populations with different household incomes, racial and/or ethnic profiles, and experiences. However, it should be noted that the W1 sample closely resembled the general population regarding sex, household income, and ethnicity, using the 2017 Statistics Canada census profile [36].

## Conclusions

The findings of the current study identify key ACEs and risk factors associated with vaping and vaping trajectories among adolescents and young adults – a public health concern of epidemic-size as identified by several health agencies [1–4]. An overwhelming proportion of youth who reported vaping at W1 also reported vaping over time, emphasizing the need for early prevention. The factors identified in the current study can help inform programs and strategies to prevent vaping among adolescents and young adults and help identify youth that may be targeted for vaping interventions. The findings on ACEs provide further evidence on the need to advocate for children’s rights to protection from childhood adversities that may be associated with harmful substance use and associated lifelong consequences.

## Data Availability

The datasets analyzed during the current study are not publicly available due to the sensitive nature of the data and privacy and confidentially guidelines which states the data must be housed in a secured lab and cannot be made publicly available, but are available from the corresponding author on reasonable request.

## Abbreviations

ACEs: Adverse childhood experiences
US: United States of America
IPV: Intimate partner violence
WE Study: Well-Being and Experiences Study
W1: Wave 1
W2: Wave 2
CPO: Child protective organization
OR: Odds ratio
AOR: Adjusted odds ratio
CI: Confidence interval

## References

[1] Gottlieb S. Statement from FDA Commissioner Scott Gottlieb, M.D., on proposed new steps to protect youth by preventing access to flavored tobacco products and banning menthol in cigarettes. 2018. Available from: https://www.fda.gov/news-events/press-announcements/statement-fda-commissioner-scott-gottlieb-md-proposed-new-steps-protect-youth-preventing-access. Cited 2021 Feb 12.

[2] National Center for Chronic Disease Prevention and Health Promotion (US) Office on Smoking and Health. E-cigarette use among youth and young adults: a report of the Surgeon General. Atlanta: Centers for Disease Control and Prevention; 2016. 30869850

[3] Public Health Agency of Canada (2020). Statement from the Council of Chief Medical Officers of Health on Nicotine Vaping in Canada.

[4] US Department of Health and Human Services (2018). Surgeon general releases advisory on E-cigarette epidemic among youth.

[5] Gentzke AS, Creamer M, Cullen KA, Ambrose BK, Willis G, Jamal A (2019). Vital signs: Tobacco product use among middle and high school students — United States, 2011–2018. MMWR Morb Mortal Wkly Rep.

[6] Wang TW, Gentzke AS, Sharapova S, Cullen KA, Ambrose BK, Jamal A (2018). Tobacco product use among middle and high school students — United States, 2011–2017. MMWR Morb Mortal Wkly Rep.

[7] Hammond D, Reid JL, Rynard VL, Fong GT, Cummings KM, McNeill A, et al. Prevalence of vaping and smoking among adolescents in Canada, England, and the United States: repeat national cross sectional surveys. BMJ. 2019;365. 10.1136/bmj.l2219 Cited 2021 Apr 16. 10.1136/bmj.l2219 PMC6582265 31221636

[8] Dai H, Leventhal AM (2019). Prevalence of e-cigarette use among adults in the United States, 2014–2018. JAMA.

[9] Kwon E, Seo D-C, Lin H-C, Chen Z (2018). Predictors of youth e-cigarette use susceptibility in a U.S. nationally representative sample. Addict Behav.

[10] Sawdey MD, Day HR, Coleman B, Gardner LD, Johnson SE, Limpert J (2019). Associations of risk factors of e-cigarette and cigarette use and susceptibility to use among baseline PATH study youth participants (2013–2014). Addict Behav.

[11] Barrington-Trimis JL, Berhane K, Unger JB, Cruz TB, Huh J, Leventhal AM (2015). Psychosocial factors associated with adolescent electronic cigarette and cigarette use. Pediatrics.

[12] Babineau K, Taylor K, Clancy L (2015). Electronic cigarette use among Irish youth: a cross sectional study of prevalence and associated factors. Niaura R, editor. PLoS One.

[13] Elgar FJ, Donnelly PD, Michaelson V, Gariépy G, Riehm KE, Walsh SD (2018). Corporal punishment bans and physical fighting in adolescents: an ecological study of 88 countries. BMJ Open.

[14] Riehm KE, Young AS, Feder KA, Krawczyk N, Tormohlen KN, Pacek LR, et al. Mental health problems and initiation of e-cigarette and combustible cigarette use. Pediatrics. 2019;144(1) Available from: https://www.ncbi.nlm.nih.gov/pmc/articles/PMC6615573/. Cited 2021 Mar 5. 10.1542/peds.2018-2935 PMC6615573 31160343

[15] Kong G, Kuguru KE, Krishnan-Sarin S (2017). Gender differences in U.S. adolescent e-cigarette use. Curr Addict Rep.

[16] Felitti VJ, Anda RF, Nordenberg D, Williamson DF, Spitz AM, Edwards VJ (1998). Relationship of childhood abuse and household dysfunction to many of the leading causes of death in adults: the adverse childhood experiences (ACE) study. Am J Prev Med.

[17] Vander Weg MW (2011). Adverse childhood experiences and cigarette smoking: the 2009 Arkansas and Louisiana behavioral risk factor surveillance systems. Nicotine Tob Res.

[18] Duke NN (2018). Adolescent adversity and concurrent tobacco, alcohol, and marijuana use. Am J Health Behav.

[19] Mersky JP, Topitzes J, Reynolds AJ (2013). Impacts of adverse childhood experiences on health, mental health, and substance use in early adulthood: a cohort study of an urban, minority sample in the U.S. Child Abus Negl.

[20] Allem JP, Soto DW, Baezconde-Garbanati L, Unger JB (2015). Adverse childhood experiences and substance use among Hispanic emerging adults in Southern California. Addict Behav.

[21] Afifi TO, Henriksen CA, Asmundson GJG, Sareen J (2012). Childhood maltreatment and substance use disorders among men and women in a nationally representative sample. Can J Psychiatr.

[22] Shin SH, Conley D, Ksinan Jiskrova G, Wills TA (2019). Adverse childhood experiences and e-cigarette use during young adulthood. Am J Addict.

[23] Melka A, Chojenta C, Holliday E, Loxton D (2019). Adverse childhood experiences and electronic cigarette use among young Australian women. Prev Med (Baltim).

[24] Martinasek MP, Wheldon CW, Parsons CA, Bell LA, Lipski BK. Understanding adverse childhood experiences as predictors of cigarette and e-cigarette use. Am J Prev Med. 2021; In press. Available from: https://www-clinicalkey-com.uml.idm.oclc.org/#!/content/playContent/1-s2.0-S0749379721000842?scrollTo=%23hl0001204. Cited 2021 Apr 16. 10.1016/j.amepre.2021.01.004 33781621

[25] Ofuchi T, Zaw AMM, Thepthien BO. Adverse childhood experiences and prevalence of cigarette and e-cigarette use among adolescents in Bangkok, Thailand. Asia-Pacific J Public Health. 2020;32(8):398–405. Available from: http://journals.sagepub.com/doi/10.1177/1010539520962956. Cited 2021 Mar 5. 10.1177/1010539520962956 33025794

[26] Afifi TO, Salmon S, Garcés I, Struck S, Fortier J, Taillieu T (2020). Confirmatory factor analysis of adverse childhood experiences (ACEs) among a community-based sample of parents and adolescents. BMC Pediatr.

[27] Afifi TO, Taillieu T, Salmon S, Davila IG, Stewart-Tufescu A, Fortier J (2020). Adverse childhood experiences (ACEs), peer victimization, and substance use among adolescents. Child Abuse Negl.

[28] Williams L, Clements-Nolle K, Lensch T, Yang W (2020). Exposure to adverse childhood experiences and early initiation of electronic vapor product use among middle school students in Nevada. Addict Behav Rep.

[29] Westling E, Rusby JC, Crowley R, Light JM (2017). Electronic cigarette use by youth: prevalence, correlates, and use trajectories from middle to high school. J Adolesc Health.

[30] Bernstein DP, Fink L (1998). Childhood trauma questionnaire: a retrospective self-report.

[31] Walsh CA, MacMillan HL, Trocmé N, Jamieson E, Boyle MH (2008). Measurement of victimization in adolescence: development and validation of the childhood experiences of violence questionnaire. Child Abuse Negl.

[32] Statistics Canada (2019). Canadian Tobacco Alcohol and Drugs (CTADS) survey: 2017 summary. Ctads 2017.

[33] Soneji S, Barrington-Trimis JL, Wills TA, Leventhal AM, Unger JB, Gibson LA (2017). Association between initial use of e-cigarettes and subsequent cigarette smoking among adolescents and young adults a systematic review and meta-analysis. JAMA Pediatr.

[34] Al-Hamdami M, Hopkins DB, Davidson M (2021). The 2020-2021 youth and young adult vaping project.

[35] McLennan JD, MacMillan HL (2016). Routine primary care screening for intimate partner violence and other adverse psychosocial exposures: what’s the evidence?. BMC Fam Pract.

[36] Statistics Canada (2017). Winnipeg, CY, Manitoba and Canada (table).

